# Factors Affecting Psychological Well-Being, Depression, Anxiety, and Stress Among Dental Students in Delhi and the National Capital Region: A Cross-Sectional Study

**DOI:** 10.7759/cureus.70474

**Published:** 2024-09-29

**Authors:** Shakila Mahesh, Kruthiventi Hemalata, Alpa Gupta

**Affiliations:** 1 Microbiology, Manav Rachna Dental College, Faridabad, IND; 2 Dentistry, Manav Rachna International Institute of Research and Studies, Faridabad, IND

**Keywords:** anxiety, dass-21 scale, mental health status, postgraduate (pg), psychological well-being, stress, undergraduate (ug), university students

## Abstract

Introduction

Research has shown that mental health issues are prevalent among university students and healthcare professionals. Dental practitioners are particularly at risk due to the demanding nature of their profession. Our study aims to survey dental students and faculty to assess their psychological well-being and identify key stressors and potential interventions.

Methodology

A cross-sectional study examined the psychological well-being, depression, anxiety, and stress of undergraduate (UG) and postgraduate (PG) dental students and faculty from the School of Dental Sciences. A questionnaire was distributed via email and WhatsApp to 350 participants. The questionnaire contained a unique set of items for each study group related to factors affecting psychological well-being among students, and a separate questionnaire was used to assess depression, anxiety, and stress (DASS-21) in the same population.

Results

A cross-sectional study of 350 dental students and educators found no significant changes in depression, anxiety, and stress (DASS) levels between age groups. Females, however, reported greater levels of these difficulties than men (D=71.5%, A=71.9%, S=76.7% for females vs. D=28.5%, A=28.1%, S=23.3% for men, with p-values for D=0.014, A=0.001, and S=0.031). Overall, 67.3% of UG students study away from home and live in hostels or flats, with academic and financial demands (30.6% and 33.5%, p-values: 0.014 and 0.098, respectively). Many people find their workload unpleasant (40.8%, p-value=0.143) and feel overwhelmed by academic obligations (47.9%, p-value=0.002), with 20%-25% worried about examinations, failure, and balancing well-being with academics (p-values: 0.001, 0.006, and 0.002, respectively). In total, 57.1% suffer severe social isolation (p-value=0.045), and more than 40% have mental health issues (p=0.003). While the mentor-mentee program is rated as helpful (p-value=0.68), 35% of students are ignorant of mental health options (p-value=0.006). Financial concerns are a big worry, with 38.7% seeking assistance, and second- and third-year students suffer more challenges than first-year students. Overall, 41.2% of instructors are perceived as extremely accessible, whereas 34.3% and 31.4% feel that scheduled breaks are required due to stress (38.2%, p=0.053). Most instructors (51.4%) encourage counseling and interact with students (76.5%), while 77.1% discuss mental health. Among the PGs, 74.2% of participants are content with the college's amenities, but 90.3% are stressed because of the PG seat departing bond, demonstrating widespread concern over this issue. To cope, 22.6% seek help from loved ones, while 19.4% pursue hobbies, emphasizing the value of personal support networks and leisure activities.

Conclusion

The study found that females perceive more stress than males. Most UG students live with supportive families and only experience academic helplessness on rare occasions. First-year students find academic effort more demanding than their peers at higher educational levels. Overall academic load gradually decreases from the first to the third year but rises significantly in the final year before declining during internships. As academic load increases, students tend to isolate themselves socially. Engaging in leisure activities can help mitigate the negative effects of stress on physical and psychological health. Faculty members recommend college counseling services to help students cope with stress.

## Introduction

Research has indicated that mental health problems are among the most prevalent health issues experienced by university students [1,2]. In 2017, WHO reported that approximately 322 million individuals, i.e., almost 4% of the global population, suffer from various forms of depression [3].

The clinical performance of healthcare professionals depends on the psychological well-being of that individual. It was noted that the performance of healthcare providers is dependent on their psychological well-being. Negative effects were seen in the care given by the caregivers who show negative/depressive symptoms. Caregivers with a positive mindset or those who are not depressed perform better and provide much more effective care [4].

Among the various healthcare providers, dental practitioners are particularly known to be vulnerable to mental health risks, primarily due to the unique demands of the profession itself. It was noted that there is a higher prevalence of stress, anxiety, and burnout among dental students [5,6]. A meta-analysis in 2021 showed that a prevalence of 29% of dental students show depressive symptoms [7].

This can be due to the fact that the dental curriculum in itself is very demanding. It demands not only theoretical proficiency but also technical and clinical efficiency within a restricted period. All these factors are major stressors that can lead to mental health challenges [8,9]. A study conducted in 2010 reported that even the faculty members who are responsible for mentoring the students are at risk of experiencing psychological disorders as they try to navigate between balancing their clinical practice and academic duties [10].

Despite these challenges being recognized worldwide, there is a paucity of data specifically addressing the psychological well-being of dental students and faculty within the academic institution. The existing studies focus either on faculty or students separately and do not provide a comprehensive, broad, college-wide perspective. To address this gap, our study aims to conduct a detailed survey of both dental students and faculty to assess their psychological well-being and identify key stressors and potential interventions.

## Materials and methods

Study design

A cross-sectional study has been designed to assess depression, anxiety, and stress (DASS-21) and the psychological well-being of the students in the dental facility. Data were collected through an electronic self-administered questionnaire prepared in English, which was sent to different colleges via email and WhatsApp. The survey was carried out from July to August 2024.

Participants

Sample size estimation was done using GPower software 3.0. At a 95% confidence interval and 80% power of the study with an effect size of 0.8, a final sample size of 350 participants was selected. The sampling method used was the snowball sampling method.

The inclusion criteria include undergraduate (UG) and postgraduate (PG) students currently pursuing a bachelor's degree in dentistry and teachers teaching at dental colleges across Delhi and the National Capital Region (NCR). The people who agreed to participate in the study volunteered. The exclusion criteria included inadequate responses and refusal to provide consent.

Study instruments

Two sets of questionnaires were created. The first was designed to assess depression, anxiety, and stress levels (DASS-21) among students and instructors [11]. The second set of questionnaires included a total of 71 items, 22 for UGs, 22 for PGs, and 27 for faculty, all of which addressed issues influencing student well-being. The questionnaire was developed in collaboration with the Department of Psychology. Nine experts in the area created the questionnaire based on previous research [12,13] and validated it for content validity and internal consistency using the content validity index (CVI) and Cronbach's alpha. The item-level content validity index (I-CVI) and universal content validity index (U-CVI) scores demonstrated acceptable and good content validity after making appropriate revisions to the questionnaire. Additionally, the questionnaire has a Cronbach's alpha score of 0.78 for nine experts, indicating adequate internal consistency and reliability. A pilot study of 15 individuals, five from each category (faculty, PGs, and UGs), was done to assess face validity. The Kappa value was determined and found to be 0.8, indicating significant agreement among participants.

A Google form was created, and the link was shared online with all organizations. After completing the first section, the form sends them to the desired category: faculty/PG student/UG student.

Ethical consideration

The study adhered to the study protocol, which was proposed to the ethical committee and approved by the Institutional Ethical Committee. Informed consent was obtained before the initiation of the study. Anonymity and confidentiality were maintained for all participants throughout the study.

Statistical analysis

The data were coded and recorded in a Microsoft Excel sheet (Microsoft Corporation, Redmond, Washington). The total data were distributed meaningfully and presented as individual tables. The data analysis was done using the IBM SPSS Statistics for Windows, Version 27 (Released 2020; IBM Corp., Armonk, New York). Descriptive statistics such as mean, standard deviation, and percentage were used along with the chi-square test and one-way ANOVA, followed by the post-hoc Bonferroni for intergroup comparisons of means. Statistical significance was set at a value of p<0.05.

## Results

Demographic distribution of the participants

A cross-sectional study was conducted among university students and faculty (N=350), with 294 UG participants (male=67, female=217), 31 PG participants (male=3, female=28), and 34 faculty (male=4, female=30). Most participants have 1-10 lakh per annum income (UG=49.6% and PG=41.9%). The data have been divided into three categories: UG students, PG students, and faculty (Table 1).

**Table 1:** Demographic distribution of the study population PG: postgraduate; UG: undergraduate

		Faculty (n/%)	PG (n/%)	UG (n/%)
Gender	Males	4 (11.8%)	3(9.7%)	67 (23.6%)
	Females	30 (88.2%)	28 (90.3%)	217 (76.4%)
Year of study	First	-	14 (45.2%)	77 (27.1%)
	Second	-	11 (35.5%)	53 (18.7%)
	Third	-	6 (19.4%)	42 (14.8%)
	Fourth	-	-	70 (24.6%)
	Intern	-	-	42 (14.8%)
Family income	1-10 lakh/annum	-	13 (41.9%)	141 (49.6%)
	11-20 lakh/annum	-	11 (35.5%)	72 (25.4%)
	21-30 lakh/annum	-	6 (19.4%)	41 (14.4%)
	41-50 lakh/annum	-	1 (3.2%)	18 (6.3%)
	51 lakh or more/annum	-	-	12 (4.2%)
Primary schooling	Rural	-	-	14 (4.9%)
	Semi-urban	-	-	21 (7.4%)
	Urban	-	-	249 (87.7%)

Interpretation of DASS-21 scores for the prevalence of psychological distress

Table 2 shows the distribution of the scores of depression, anxiety, and stress among the participants. For depression, most of the individuals fall into the Moderate category (30.9%). A relatively low percentage (8.0%) of the study participants are classified as Extremely Severe. On assessing anxiety, the results are notably different. A significant 42.0% of the participants are classified as having Extremely Severe anxiety, indicating a major concern within this population. In contrast, when analyzing stress scores, the majority of individuals are in the Normal category, comprising 57.1% of the sample. This suggests that stress levels are relatively lower in this group compared to anxiety, with only 9.7% of participants classified as Extremely Severe.

**Table 2:** DASS-21 scores frequency for prevalence of psychological distress

Scores	Depression		Anxiety		Stress	
	N	%	N	%	N	%
Normal	94	26.9	69	19.7	200	57.1
Mild	64	18.3	9	2.6	37	10.6
Moderate	108	30.9	78	22.3	44	12.6
Severe	56	16	47	13.4	35	10
Extremely Severe	28	8	147	42	34	9.7

DASS-21 scores across different demographics

Table 3 shows that there are no significant differences in depression, anxiety, or stress, indicating that levels remain similar across ages. However, there are significant gender disparities, with females reporting higher levels of depression, anxiety, and stress than males (females: D=71.5%, A=71.9%, S=76.7%; males: D=28.5%, A=28.1%, S=23.3%), and these differences are statistically significant (p-values: D=0.014, A=0.001, S=0.031). There are no significant differences in DASS scores across academic or professional categories; nevertheless, students have greater percentages in all three areas than the faculty and PG groups, although these differences are not statistically significant.

**Table 3:** Depression, anxiety, and stress (DASS-21) scores according to variables *p-value significant at p<0.05; the chi-square test was applied

Variables		Depression		Anxiety		Stress	
		N	%	N	%	N	%
Total population		256	73.1%	281	80.3%	150	42.9%
Age group	18-24	193	75.4%	205	73.0%	110	73.3%
	25-34	42	16.4%	48	17.1%	25	16.7%
	35-44	16	6.3%	21	7.5%	12	8.0%
	45-54	4	1.6%	6	2.1%	2	1.3%
	55-64	1	0.4%	1	0.4%	1	0.7%
	p-value	0.071		0.071		0.762	
Gender	Female	183	71.5%	202	71.9%	115	76.7%
	Male	73	28.5%	79	28.1%	35	23.3%
	Prefer not to say	0	0	0	0	0	0
	p-value	0.014*		0.001*		0.031*	
Category	Faculty	23	9.0%	29	10.3%	15	10.0%
	PG	18	7.0%	26	9.3%	12	8.0%
	UG	215	84.0%	226	80.4%	123	82.0%
	p-value	0.063		0.783		0.886	

Factors affecting the psychological well-being of UG dental students

Although not statistically significant, the majority of UGs (67.3%) study outside of their home state and live in hostels or apartments. Many students have supportive to extremely supportive families, although some experience academic pressure and financial strain, with 30.6% and 33.5% reporting these concerns, respectively (p-values: 0.014 and 0.098). The majority of participants find their academic workload stressful (40.8%, p=0.143) and feel helpless in the face of academic obligations (47.9%, p=0.002). While the majority are undecided regarding the effectiveness of tests, the influence of fear of failure, and reconciling personal well-being with academics, 20%-25% agree with these concerns (p-values: 0.001, 0.006, and 0.002). The data demonstrate high social isolation (57.1%, p=0.045), and although most students are not diagnosed with mental health disorders, over 40% report diagnoses such as anxiety, ADHD, depression, or eating disorders (p=0.003). While the mentor-mentee program is rated as effective (p-value=0.68), more than 40% are aware of college mental health resources, while 35% are clueless (p-value=0.006). Many students struggle with mental health issues, social isolation, and managing academics. Additionally, assistance from family, friends, and professors is essential (Table 4).

**Table 4:** Factors affecting the psychological well-being of undergraduate dental students based on the years of study *p-value significant at p<0.05; the chi-square test was applied

S. No.	Questions	Answer	I (n/%)	II (n/%)	III (n/%)	IV (n/%)	Intern (n/%)	Total (n/%)	p-value
1	College in the state you belong	No	52 (67.5%)	35 (66%)	31 (73.8%)	47 (67.1%)	26 (61.9%)	191 (67.3%)	0.844
		Yes	25 (32.5%)	18 (34%)	11 (26.2%)	23 (32.9%)	16 (38.1%)	93 (32.7%)	
2	Where do you reside	Day scholar	0	0	0	1 (1.4%)	0	1 (0.4%)	*0.003
		Guardian	0	0	0	1 (1.4%)	0	1 (0.4%)	
		Hostel	29 (37.7%)	21 (39.6%)	12 (28.6%)	3 (4.3%)	3 (7.1%)	68 (23.9%)	
		On rent	0	0	0	1 (1.4%)	0	1 (0.4%)	
		Apartment	9 (11.7%)	2 (3.8%)	3 (7.1%)	16 (22.9%)	6 (14.3%)	36 (12.7%)	
		Parent	37 (48.1%)	29 (54.7%)	25 (59.5%)	43 (61.4%)	30 (71.4%)	164 (57.7%)	
		Paying guest	0	0	0	1 (1.4%)	0	1 (0.4%)	
		PG	1 (1.3%)	0	1 (2.4%)	1 (1.4%)	2 (4.8%)	5 (1.8%)	
		Relatives	1 (1.3%)	1 (1.9%)	1 (2.4%)	3 (4.3%)	1 (2.4%)	7 (2.5%)	
3	Overall supportive nature of family	Extremely unsupportive	2 (2.6%)	4 (7.5%)	2 (4.8%)	3 (4.3%)	2 (4.8%)	13 (4.6%)	0.102
		Unsupportive	2 (2.6%)	0	1 (2.4%)	2 (2.9%)	1 (2.4%)	6 (2.1%)	
		Neutral	15 (19.5%)	9 (17%)	1 (2.4%)	4 (5.7%)	3 (7.1%)	32 (11.3%)	
		Supportive	30 (39%)	22 (41.5%)	16 (38.1%)	20 (28.6%)	13 (31%)	101 (35.6%)	
		Extremely supportive	28 (36.4%)	18 (34%)	22 (52.4%)	41 (58.6%)	23 (54.8%)	132 (46.5%)	
4	Pressure on academics from family	Always	5 (6.50%)	6 (11.30%)	0	2 (2.90%)	2 (4.80%)	15 (5.30%)	*0.014
		Often	9 (11.70%)	9 (17%)	4 (9.50%)	4 (5.70%)	1 (2.40%)	27 (9.50%)	
		Sometimes	21 (27.30%)	19 (35.80%)	15 (35.70%)	19 (27.10%)	13 (31%)	87 (30.60%)	
		Supportive	30 (39%)	8 (15.10%)	12 (28.60%)	18 (25.70%)	11 (26.20%)	79 (27.80%)	
		Extremely supportive	12 (15.60%)	11 (20.80%)	11 (26.20%)	27 (38.60%)	15 (35.70%)	76 (26.80%)	
5	Financial strain in family	Always	8 (10.4%)	8 (15.1%)	1 (2.4%)	6 (8.6%)	4 (9.5%)	27 (9.5%)	0.098
		Often	12 (15.6%)	7 (13.2%)	5 (11.9%)	7 (10%)	0	31 (10.9%)	
		Sometimes	30 (39%)	19 (35.8%)	12 (28.6%)	21 (30%)	13 (31%)	95 (33.5%)	
		Supportive	20 (26%)	9 (17%)	16 (38.1%)	22 (31.4%)	12 (28.6%)	79 (27.8%)	
		Extremely supportive	7 (9.1%)	10 (18.9%)	8 (19%)	14 (20%)	13 (31%)	52 (18.3%)	
6	Overall load of academics	Easy	1 (1.3%)	0	1 (2.4%)	1 (1.4%)	1 (2.4%)	4 (1.4%)	0.143
		Manageable	34 (44.2%)	13 (24.5%)	17 (40.5%)	28 (40%)	22 (52.4%)	114 (40.1%)	
		Stressful	33 (42.9%)	23 (43.4%)	20 (47.6%)	28 (40%)	12 (28.6%)	116 (40.8%)	
		Very easy	0	0	0	1 (1.4%)	1 (2.4%)	2 (0.7%)	
		Very stressful	9 (11.7%)	17 (32.1%)	4 (9.5%)	12 (17.1%)	6 (14.3%)	48 (16.9%)	
7	Helpless by academic demands	Always	3 (3.90%)	10 (18.90%)	2 (4.80%)	12 (17.10%)	3 (7.10%)	30 (10.60%)	*0.002
		Never	1 (1.30%)	0	2 (4.80%)	1 (1.40%)	2 (4.80%)	6 (2.10%)	
		Often	26 (33.80%)	20 (37.70%)	15 (35.70%)	6 (8.60%)	14 (33.30%)	81 (28.50%)	
		Rarely	12 (15.60%)	3 (5.70%)	3 (7.10%)	7 (10%)	6 (14.30%)	31 (10.90%)	
		Sometimes	35 (45.50%)	20 (37.70%)	20 (47.60%)	44 (62.90%)	17 (40.50%)	136 (47.90%)	
8	Skills and knowledge assessed appropriately by exams	Strongly disagree	0	6 (11.30%)	0	1 (1.40%)	4 (9.40%)	11 (3.90%)	*0.001
		Disagree	12 (15.6%)	9 (17%)	2 (4.80%)	3 (4.30%)	10 (23.80%)	36 (12.70%)	
		Neutral	38 (49.40%)	20 (37.70%)	20 (47.60%)	40 (57.10%)	13 (31%)	131 (46.10%)	
		Agree	22 (28.60%)	12 (22.60%)	14 (33.30%)	21 (30%)	8 (19%)	77 (27.10%)	
		Strongly agree	5 (6.50%)	6 (11.30%)	6 (14.30%)	5 (7.10%)	7 (16.70%)	29 (10.20%)	
9	Academic performance affected by fear of failure	Strongly disagree	2 (2.60%)	6 (11.30%)	3 (7.10%)	4 (5.70%)	7 (16.70%)	22 (7.70%)	*0.006
		Disagree	11 (14.30%)	8 (15.10%)	13 (31%)	16 (22.90%)	7 (16.70%)	55 (19.40%)	
		Neutral	35 (45.50%)	10 (18.90%)	16 (38.10%)	29 (41.40%)	14 (33.30%)	104 (36.60%)	
		Agree	23 (29.90%)	23 (43.40%)	9 (21.40%)	15 (21.40%)	7 (16.70%)	77 (27.10%)	
		Strongly agree	6 (7.80%)	6 (11.30%)	1 (2.40%)	6 (8.60%)	7 (16.70%)	26 (9.20%)	
10	Difficulty balancing personal well-being and academics	Strongly disagree	0	3 (5.70%)	5 (11.90%)	3 (4.30%)	3 (4.30%)	17 (6%)	*0.002
		Slightly disagree	13 (16.90%)	8 (15.10%)	9 (21.40%)	10 (14.30%)	10 (14.30%)	46 (16.20%)	
		Neutral	42 (54.50%)	20 (37.70%)	15 (35.70%)	37 (52.90%)	37 (52.90%)	130 (45.80%)	
		Agree	14 (18.20%)	9 (17%)	11 (26.20%)	19 (27.10%)	19 (27.10%)	60 (21.10%)	
		Strongly agree	8 (10.40%)	13 (24.50%)	2 (4.80%)	1 (1.40%)	1 (1.40%)	31 (10.90%)	
11	Library hours support study	No	9 (11.70%)	10 (18.90%)	0	14 (20%)	6 (14.30%)	39 (13.70%)	0.088
		Somewhat	19 (24.70%)	17 (32.10%)	10 (23.80%)	17 (24.30%)	13 (31%)	76 (26.80%)	
		Yes	49 (63.60%)	26 (49.10%)	32 (76.20%)	39 (55.70%)	23 (54.80%)	169 (59.50%)	
12	College has sufficient measures to prevent ragging	No	7 (9.10%)	8 (15.10%)	3 (7.10%)	13 (18.60%)	9 (21.40%)	40 (14.10%)	0.288
		Unsure	17 (22.10%)	12 (22.60%)	7 (16.70%)	8 (11.40%)	6 (14.30%)	50 (17.60%)	
		Yes	53 (68.80%)	33 (62.30%)	32 (76.20%)	49 (70%)	27 (64.30%)	194 (68.30%)	
13	Pressure placed by finances on you	Extremely	5 (6.50%)	10 (18.90%)	4 (9.50%)	11 (15.70%)	2 (4.80%)	32 (11.30%)	0.069
		Significantly	20 (26%)	7 (13.20%)	8 (19%)	12 (17.10%)	5 (11.90%)	52 (18.30%)	
		Moderately	31 (40.30%)	15 (28.30%)	16 (38.10%)	26 (37.10%)	19 (45.20%)	107 (37.70%)	
		Slightly	10 (13%)	10 (18.90%)	13 (31%)	10 (14.30%)	9 (21.40%)	52 (18.30%)	
		Not stressful	11 (14.30%)	11 (20.80%)	1 (2.40%)	11 (15.70%)	7 (16.70%)	41 (14.40%)	
14	How often do you experience social isolation	Always	6 (7.80%)	7 (13.20%)	4 (9.50%)	10 (14.30%)	4 (9.50%)	4 (9.50%)	*0.045
		Never	6 (7.80%)	1 (1.90%)	1 (2.40%)	8 (11.40%)	2 (4.80%)	2 (4.80%)	
		Often	18 (23.40%)	17 (32.10%)	18 (42.90%)	12 (17.10%)	7 (16.70%)	7 (16.70%)	
		Rarely	6 (7.80%)	9 (17%)	7 (16.70%)	9 (12.90%)	5 (11.90%)	5 (11.90%)	
		Sometimes	41 (53.20%)	19 (35.80%)	12 (28.60%)	31 (44.30%)	24 (35.80%)	24 (57.10%)	
15	Ever had a diagnosis for mental health conditions	Anxiety	10 (13%)	12 (22.60%)	8 (19%)	11 (15.70%)	10 (23.80%)	51 (18%)	*0.003
		Attention -deficit/hyperactivity disorder (ADHD)	6 (7.80%)	2 (3.80%)	1 (2.40%)	1 (1.40%)	1 (2.40%)	11 (3.90%)	
		Depression	7 (9.10%)	9 (17%)	3 (7.10%)	3 (4.30%)	2 (4.80%)	24 (8.50%)	
		Eating disorders	0	2 (3.80%)	7 (16.70%)	1 (1.40%)	2 (4.80%)	12 (4.20%)	
		None	52 (67.50%)	24 (45.30%)	22 (52.40%)	51 (72.90%)	26 (61.90%)	175 (61.60%)	
		Others	2 (2.60%)	4 (7.50%)	1 (2.40%)	3 (4.30%)	1 (2.40%)	11 (3.90%)	
16	Level of support from faculty/mentors	Extremely unsupportive	1 (1.30%)	8 (15.10%)	0	7 (10%)	4 (9.50%)	20 (7%)	*0.002
		Unsupportive	8 (10.40%)	6 (11.30%)	3 (7.10%)	5 (7.10%)	3 (7.10%)	25 (8.80%)	
		Neutral	32 (41.60%)	20 (37.70%)	19 (45.20%)	30 (42.90%)	18 (42.90%)	119 (41.90%)	
		Supportive	29 (37.70%)	15 (28.30%)	16 (38.10%)	15 (21.40%)	4 (9.50%)	79 (27.80%)	
		Extremely supportive	7 (9.10%)	4 (7.50%)	4 (9.50%)	13 (18.60%)	13 (31%)	41 (14.40%)	
17	College offers mentor-mentee program	No	5 (6.50%)	1 (1.90%)	7 (16.70%)	3 (4.30%)	1 (2.40%)	17 (6%)	0.114
		Unsure	6 (7.80%)	6 (11.30%)	3 (7.10%)	5 (7.10%)	2 (4.80%)	22 (7.70%)	
		Yes	66 (85.70%)	46 (86.80%)	32 (76.20%)	62 (88.60%)	39 (92.90%)	245 (86.30%)	
18	How effective is having a mentor and meeting regularly	Neutral	25 (32.50%)	18 (34%)	13 (31%)	21 (30%)	13 (31%)	90 (31.70%)	0.68
		Somewhat effective	23 (29.90%)	16 (30.20%)	12 (28.60%)	17 (24.30%)	7 (16.70%)	75 (26.40%)	
		Somewhat ineffective	3 (3.90%)	4 (7.50%)	0	6 (8.60%)	2 (4.80%)	15 (5.30%)	
		Very effective	23 (29.90%)	14 (26.40%)	13 (31%)	24 (34.30%)	17 (40.50%)	91 (32%)	
		Very ineffective	3 (3.90%)	1 (1.90%)	4 (9.50%)	2 (2.90%)	3 (7.10%)	13 (4.60%)	
19	Ease of accessing mental assistance	Neutral	47 (61%)	23 (43.40%)	23 (54.80%)	42 (60%)	24 (57.10%)	159 (56%)	0.218
		Somewhat comfortable	12 (15.60%)	15 (28.30%)	6 (14.30%)	9 (12.90%)	5 (11.90%)	47 (16.50%)	
		Somewhat uncomfortable	8 (10.40%)	6 (11.30%)	4 (9.50%)	7 (10%)	4 (9.50%)	29 (10.20%)	
		Very comfortable	4 (5.20%)	5 (9.40%)	9 (21.40%)	5 (7.10%)	5 (11.90%)	28 (9.90%)	
		Very uncomfortable	6 (7.80%)	4 (7.50%)	0	7 (10%)	4 (9.50%)	21 (7.40%)	
20	Know about mental health services in college	No	34 (44.20%)	25 (47.20%)	15 (35.70%)	42 (60%)	18 (42.90%)	15 (35.70%)	*0.011
		Unsure	27 (35.10%)	9 (17%)	7 (16.70%)	12 (17.10%)	10 (23.80%)	7 (16.70%)	
		Yes	16 (20.80%)	19 (35.80%)	20 (47.60%)	16 (22.90%)	14 (33.30%)	20 (47.60%)	
21	Accessibility of support services in college	Accessible	10 (13%)	2 (3.80%)	5 (11.90%)	7 (10%)	6 (14.30%)	30 (10.60%)	*0.006
		Neutral	50 (64.90%)	25 (47.20%)	26 (61.90%)	43 (61.40%)	23 (54.80%)	167 (58.80%)	
		Somewhat accessible	8 (10.40%)	7 (13.20%)	8 (19%)	5 (7.10%)	0	28 (9.90%)	
		Somewhat inaccessible	6 (7.80%)	7 (13.20%)	2 (4.80%)	4 (5.70%)	7 (16.70%)	26 (9.20%)	
		Very inaccessible	3 (3.90%)	12 (22.60%)	1 (2.40%)	11 (15.70%)	6 (14.30%)	33 (11.60%)	
22	Quality of support services in college	Average	40 (51.90%)	32 (60.40%)	30 (71.40%)	39 (55.70%)	25 (59.50%)	166 (58.50%)	*0.007
		Excellent	5 (6.50%)	3 (5.70%)	4 (9.50%)	7 (10%)	7 (16.70%)	26 (9.20%)	
		Good	26 (33.80%)	5 (9.40%)	7 (16.70%)	10 (14.30%)	6 (14.30%)	54 (19%)	
		Poor	2 (2.60%)	6 (11.30%)	1 (2.40%)	5 (7.10%)	1 (2.40%)	15 (5.30%)	
		Very Poor	4 (5.20%)	7 (13.20%)	0	9 (12.90%)	3 (7.10%)	23 (8.10%)	

Factors affecting the psychological well-being of PG dental students

According to the study, 74.2% of participants are content with the college's amenities, but 90.3% are stressed because of the PG seat departing bond, demonstrating widespread concern over this issue. To cope, 22.6% seek help from loved ones, while 19.4% pursue hobbies, emphasizing the value of personal support networks and leisure activities. The majority of respondents (67.7%) report that peer contact affects their stress levels. In terms of administrative assistance, 64.5% prefer a flexible schedule to balance academic and personal responsibilities. While 87.1% avoid using substances to cope, 38.7% feel stress and substance usage are somewhat related. Many students struggle with their thesis (90.3%) and its submission (54.8%), typically owing to financial hardship, with 38.7% seeking financial assistance from the administration. Second-year (72.7%) and third-year (83.3%) students experience greater obstacles than first-year students (28.6%), implying that thesis submission gets more difficult as students progress. Overall stress levels are high (64.5%) (Table 5).

**Table 5:** Factors affecting the psychological well-being of postgraduate students based on the year of study *p-value significant at p<0.05; the chi-square test was applied

S.No.	Question	Options	First (n/%)	Second (n/%)	Third (n/%)	Total (n/%)	p-value
1	Satisfaction with overall amenities in the college	Extremely poor	0	0	0	0	*0.039
		Poor	2 (14.30%)	0	1 (16.70%)	3 (9.70%)	
		Satisfied	10 (71.40%)	11 (100%)	2 (33.30%)	23 (74.20%)	
		Good	2 (14.30%)	0	3 (50%)	5 (16.10%)	
		Very good	0	0	0	0	
2	Stress for PG seat leave bond	No	0	2 (18.2%)	1 (16.7%)	3 (9.7%)	0.253
		Yes	14 (100%)	9 (81.8%)	5 (83.3%)	28 (90.3%)	
3	Most problems of students are with	Admin	7 (50%)	4 (36.40%)	4 (66.70%)	15 (48.40%)	0.723
		Faculty	4 (28.60%)	3 (27.30%)	1 (16.70%)	8 (25.80%)	
		PG seniors	2 (14.30%)	4 (36.40%)	1 (16.70%)	7 (22.60%)	
		Senior Resident	1 (7.10%)	0	0	1 (3.20%)	
4	Satisfaction with guide support	Adequate	12 (85.70%)	9 (81.80%)	5 (83.30%)	26 (83.90%)	0.266
		Inadequate	2 (14.30%)	2 (18.20%)	0	4 (12.90%)	
		Needs improvement	0	0	1 (16.70%)	1 (3.20%)	
5	Ways to cope with stress	Relaxation techniques	2 (14.3%)	0	0	2 (6.4%)	0.112
		Postpone stress inducing tasks	4 (28.6%)	2 (18.1%)	0	6 (19.4%)	
		Pursuing hobbies	1 (7.1%)	4 (36.4%)	1 (16.7%)	6 (19.4%)	
		Taking care of oneself	1(7.1%)	4 (36.4%)	1 (16.7%)	6 (19.4%)	
		Seeking assistance from loved ones	5 (35.7%)	0	2 (18.2%)	7 (22.6%)	
6	Interaction with peers affected stress levels	No	3 (21.40%)	3 (27.30%)	4 (66.70%)	10 (32.30%)	0.127
		Yes	11 (78.60%)	8 (72.70%)	2 (33.30%)	21 (67.70%)	
7	Worried about family reaction to your stress	No	8 (57.10%)	6 (54.50%)	4 (66.70%)	18 (58.10%)	0.886
		Yes	6 (42.90%)	5 (45.50%)	2 (33.30%)	13 (41.90%)	
8	Support mechanism to address bullying	Anti-ragging committees	8 (57.1%)	1 (9.1%)	1 (16.7%)	10 (32.3%)	0.06
		Counseling	5 (35.7%)	8 (72.7%)	5 (83.3%)	18 (58.1%)	
		Good behavior	1 (7.1%)	0	0	1 (3.2%)	
		Hotline numbers	0	2 (18.2%)	0	2 (6.5%)	
9	Marriage/relationship impacted stress levels	Adversely	2 (14.3%)	0	0	2 (6.5%)	0.42
		Help cope	4 (28.6%)	6 (54.5%)	2 (33.3%)	12 (38.7%)	
		Not applicable	8 (57.1%)	5 (45.5%)	4 (66.7%)	17 (54.8%)	
10	Support expected from admin to manage relations	Communication workshops	1 (7.1%)	2 (18.2%)	0	3 (9.7%)	0.357
		Counseling services	2 (14.3%)	2 (18.2%)	3 (50%)	7 (22.6%)	
		Flexible scheduling	11 (78.6%)	6 (54.5%)	3 (50%)	20 (64.5%)	
		Not applicable	0	1 (9.1%)	0	2 (3.2%)	
11	Substance use to cope with stress	No	12 (85.70%)	9 (81.80%)	6 (100%)	27 (87.10%)	0.553
		Yes	2 (14.30%)	2 (18.20%)	0	4 (12.90%)	
12	Connection between stress levels and substance use	Not linked	5 (35.7%)	5 (45.5%)	3 (50%)	13 (41.9%)	0.388
		Somewhat linked	4 (28.6%)	5 (45.5%)	3 (50%)	12 (38.7%)	
		Strongly linked	4 (28.6%)	0	0	4 (12.9%)	
		Workload	1 (7.1%)	1 (9.1%)	0	2 (6.5%)	
13	Is psychological well-being affected by PG	No	5 (35.70%)	2 (18.20%)	2 (33.30%)	9 (29%)	0.611
		Yes	9 (64.30%)	9 (81.80%)	4 (66.70%)	22 (71%)	
14	Challenges in thesis	No	1 (7.1%)	2 (18.2%)	0	3 (9.7%)	0.437
		Yes	13 (92.9%)	9 (81.8%)	6 (100%)	28 (90.3%)	
15	Do you face challenges in thesis submission	No	10 (71.40%)	3 (27.30%)	1 (16.70%)	14 (45.20%)	*0.026
		Yes	4 (28.60%)	8 (72.70%)	5 (83.30%)	17 (54.80%)	
16	Help expected from administration with thesis	Guidance of faculty	3 (21.4%)	2 (18.2%)	4 (66.7%)	9 (29%)	0.253
		More time	3 (21.4%)	4 (36.4%)	1 (16.7%)	8 (25.8%)	
		Removal of thesis	2 (14.3%)	0	0	2 (6.5%)	
		Financial support	6 (42.9%)	5 (45.5%)	1 (16.7%)	12 (38.7%)	
17	Appropriate reasons for your mental health	All good	4 (28.6%)	8 (72.7%)	0	0	0.752
		Issues from administration	3 (21.4%)	0	0	3 (9.7%)	
		Academic stress	3(21.4%)	3 (27.3%)	5 (83.3%)	11 (35.5%)	
		Financial issues	0	0	1 (16.7%)	1 (3.2%)	
		Relationship issues	4 (28.6%)	0	0	4 (12.9%)	
18	Challenges to seek help from a counselor	Confidentiality issues	10 (71.4%)	10 (90.9%)	4 (66.7%)	24 (77.4%)	0.59
		Impact on job prospects	0	1 (9.1%)	1 (16.7%)	2 (6.5%)	
		None	1 (7.1%)	0	0	1 (3.2%)	
		Nothing	1 (7.1%)	0	0	1 (3.2%)	
		Stigma	2 (14.3%)	0	1 (16.7%)	3 (9.7%)	
19	Overall stress levels rating	High	8 (57.1%)	9 (81.8%)	3 (50%)	20 (64.5%)	0.477
		Moderate	3 (21.4%)	2 (18.2%)	2 (33.3%)	7 (22.6%)	
		Very high	3 (21.4%)	0	1 (16.7%)	4 (12.9%)	
20	Psychological wellbeing affected by financial concerns	No	9 (64.3%)	3 (27.3%)	3 (50%)	15 (48.4%)	0.184
		Yes	5 (35.7%)	8 (72.7%)	3 (50%)	16 (51.6%)	
21	Coping with financial stress	Budgeting	9 (64.30%)	6 (54.50%)	1 (16.70%)	16 (51.60%)	*0.017
		Other	3 (21.40%)	0	4 (66.70%)	7 (22.60%)	
		Loans	2 (14.30%)	5 (45.50%)	1 (16.70%)	8 (25.80%)	
22	Effective self-care practices	Exercise	2 (14.3%)	0	0	2 (6.4%)	0.11
		Music	4 (28.6%)	2 (18.1%)	0	6 (19.4%)	
		Meditation	1 (7.1%)	4(36.4%)	1 (16.7%)	6 (19.4%)	
		Sleep	1(7.1%)	4 (36.4%)	1(16.7%)	6 (19.4%)	
		Socialize with friends	5 (35.7%)	0	2 (18.2%)	7 (22.6%)	
		Spent time with family	1 (7.1%)	1 (9.1%)	2 (18.2%)	4 (12.9%)	

Influence of faculty on the psychological well-being of UG and PG students

The data show that 41.20% of teachers perceive themselves as very approachable. Overall, 34.30% and 31.40% of faculty members believe it is somewhat very necessary for students to have designated breaks as they believe the college academic schedule (38.20%, p-value= 0.053) and faculty members themselves (51.4%; p-value= 0.247) create stress for the students.

To reduce stress, the majority of the faculty members advise counseling services from the college (51.4%) and try to engage with the students (76.5%) to alleviate their stress levels. Although the faculty had discussions with students about mental wellness (77.1%), the major chunk does not share examples to help the students overcome challenges (91.4%). In total, 51.4% of faculties believe that they can influence students' psychological well-being and would like to mentor students (38.20%) for their betterment under the mentor-mentee program. Thus, agreeing that the implementation of the mentor-mentee program (37.1%) and effective communication (37.1%) can enhance the psychological well-being of the students (Table 6).

**Table 6:** Influence of faculty on the psychological well-being of undergraduate and postgraduate students and the stress level of faculty in workplace *p-value significant at p<0.05; the chi-square test was applied

S. No.	Question	Answer	<10 Years of Teaching Experience	10-20 Years of Teaching Experience	20-30 Years of Teaching Experience	>30 Years of Teaching Experience	Total	p-value
1	Level of approachability to student	Very unapproachable	2 (11.8%)	2 (15.40%)	0	0	4 (11.80%)	0.602
		Somewhat	0	1 (7.70%)	0	0	1 (2.90%)	
		Neutral	5 (29.40%)	1 (7.70%)	0	0	6 (17.60%)	
		Somewhat approachable	5 (29.40%)	4 (30.80%)	0	0	9 (26.50%)	
		Very approachable	5 (29.40%)	6 (42.90%)	2 (100%)	2 (100%)	15 (41.20%)	
2	Students should have designated leisure breaks	Very unnecessary	4 (23.50%)	1 (7.70%)	0	0	5 (14.30%)	0.513
		Somewhat unnecessary	0	0	0	0	0	
		Neutral	3 (17.60%)	3 (23.10%)	1 (50%)	0	7 (20%)	
		Somewhat necessary	6 (35.30%)	5 (38.50%)	0	0	11 (31.40%)	
		Very necessary	4 (23.50%)	5 (35.70%)	1 (50%)	2 (100%)	13 (34.30%)	
3	College academic schedule create stress	Extremely stressful	0	0	0	0	0	0.053
		Stressful	0	2 (14.30%)	0	1 (50%)	2 (5.90%)	
		Moderately stressful	2 (11.80%)	7 (50%)	1 (50%)	1 (50%)	11 (32.40%)	
		Slightly stressful	8 (47.10%)	4 (28.60%)	1 (50%)	0	13 (38.20%)	
		Not stressful	7 (41.20%)	1 (7.10%)	0	0	8 (23.50%)	
4	Faculty members create stress	No	8 (47.10%)	7 (50%)	2 (100%)	0	17 (48.6%)	0.247
		Yes	9 (52.90%)	7 (50%)	0	2 (100%)	18 (51.4%)	
5	Have counseling services in college	No	8 (47.1%)	7 (50%)	2 (100%)	0	17 (48.6%)	0.694
		Yes	9 (52.9%)	7 (50%)	0	2 (100%)	18 (51.4%)	
6	Advised these facilities to students	No	6 (35.3%)	8 (57.1%)	2 (100%)	1 (50%)	17 (48.6%)	0.292
		Yes	11 (64.7%)	6 (42.9%)	0	1 (50%)	18 (51.4%)	
7	Students referred to counseling services in the past five years	>5	1 (5.9%)	0	0	0	1 (2.9%)	0.932
		1-3	4 (23.5%)	7 (50%)	1 (50%)	1 (50%)	13 (37.1%)	
		4-5	2 (11.8%)	1 (7.1%)	0	0	3 (8.6%)	
		None	10 (58.8%)	6 (42.9%)	1 (50%)	1 (50%)	18 (51.4%)	
8	Actions taken to alleviate mental stress	Additional assistance in teaching	4 (23.50%)	2 (15.40%)	1 (50%)	0	7 (20.60%)	0.653
		Engaging with them	13 (76.50%)	10 (76.90%)	1 (50%)	2 (100%)	26 (76.50%)	
		Sufficient rest and nutrition	0	1 (7.70%)	0	0	1 (2.90%)	
9	Difficulty in handling depression in students	No	9 (52.9%)	9 (64.3%)	2 (100%)	0	20 (57.1%)	0.205
		Yes	8 (47.1%)	5 (35.7%)	0	0	15 (42.9%)	
10	Ever shared examples to help students overcome challenges	No	0	3 (21.4%)	0	0	32 (91.4%)	0.178
		Yes	17 (100%)	11 (78.6%)	2 (100%)	2 (100%)	3 (8.6%)	
11	Does the college have well-defined system to handle cases of depression	No	4 (23.5%)	3 (21.4%)	1 (50%)	2 (100%)	10 (28.6%)	0.111
		Yes	13 (76.5%)	11 (78.6%)	1 (50%)	0	25 (71.4%)	
12	Extracurricular events organized in the last two years	>5	2 (11.8%)	3 (21.4%)	0	0	5 (14.3%)	0.933
		1-3	10 (58.8%)	7 (50%)	2 (100%)	2 (100%)	21 (60%)	
		4-5	1 (5.9%)	1 (7.1%)	0	0	2 (5.7%)	
		None	4 (23.5%)	3 (21.4%)	0	0	7 (20%)	
13	Important to organize extracurricular events	Very unnecessary	5 (29.40%)	0	1 (50%)	0	6 (17.60%)	0.298
		Somewhat unnecessary	0	1 (7.70%)	0	0	1 (2.90%)	
		Neutral	1 (5.90%)	0	0	0	1 (2.90%)	
		Somewhat necessary	3 (17.60%)	7 (53.80%)	1 (50%)	0	11 (32.40%)	
		Very necessary	8 (47.10%)	5 (38.50%)	0	2 (100%)	15 (44.10%)	
14	Had discussions with students about mental wellness	No	2 (11.8%)	6 (42.9%)	0	0	8 (22.9%)	0.136
		Yes	15 (88.2%)	8 (57.1%)	2 (100%)	2 (100%)	27 (77.1%)	
15	Do you effectively communicate ideas to your seniors	Never	2 (11.8%)	0	0	0	2 (5.7%)	0.547
		Rarely	0	2 (14.3%)	0	0	2 (5.7%)	
		Sometimes	6 (35.3%)	4 (28.6%)	0	1 (50%)	11 (31.4%)	
		Always	7 (41.2%)	5 (35.7%)	2 (100%)	0	14 (40%)	
		Often	2 (11.8%)	3 (21.4%)	0	1 (50%)	6 (17.1%)	
16	System to handle depression	No	4 (23.50%)	3 (23.10%)	1 (50%)	2 (100%)	10 (29.40%)	0.125
		Yes	13 (76.50%)	10 (76.90%)	1 (50%)	0	24 (70.60%)	
17	Protocol to address discrimination	No	3 (17.60%)	1 (7.70%)	0	0	4 (11.80%)	0.727
		Yes	14 (82.40%)	12 (92.30%)	2 (100%)	2 (100%)	30 (88.20%)	
18	Your stress levels at workplace	Not stressful	1 (5.90%)	2 (15.40%)	0	0	3 (8.80%)	0.244
		Slightly stressful	8 (47.10%)	2 (15.40%)	0	0	10 (29.40%)	
		Moderately stressful	5 (29.40%)	4 (30.80%)	2 (100%)	1 (50%)	12 (35.30%)	
		Stressful	3 (17.60%)	3 (23.10%)	0	0	6 (17.60%)	
		Extremely stressful	0	2 (15.40%)	0	1 (50%)	3 (8.80%)	
19	Stress management techniques used	Applied time management	0	1 (7.1%)	0	0	1 (2.9%)	0.894
		Connect with nature	1 (5.9%)	0	0	0	1 (2.9%)	
		Spiritual contemplation	0	3 (21.4%)	0	0	3 (8.6%)	
		Enjoy favorite meal	2 (11.8%)	0	0	0	2 (5.7%)	
		Exercise	2 (11.8%)	2 (14.3%)	0	0	4 (11.4%)	
		Music	1 (5.9%)	1 (7.1%)	0	1 (50%)	3 (8.6%)	
		Meditation	1 (5.9%)	0	0	0	1 (2.9%)	
		Sleep	2 (11.8%)	2 (14.3%)	0	0	4 (11.4%)	
		Socialize with friends	1 (5.9%)	1 (7.1%)	0	0	2 (5.7%)	
		Spent time with family	7 (41.2%)	4 (28.6%)	2 (100%)	1 (50%)1	4 (40%)	
20	do you have ability to influence your student psychological well-being	Strongly disagree	1 (5.9%)	1 (7.1%)	0	0	2 (5.7%)	0.481
		Disagree	0	0	0	0	0	
		Neutral	6 (35.3%)	4 (28.6%)	0	0	10 (28.6%)	
		Agree	7 (41.2%)	7 (50%)	2 (100%)	2 (100%)	18 (51.4%)	
		Strongly agree	3 (17.6%)	2 (14.3%)	0	0	5 (14.3%)	
21	Does your superiors will support to launch activities for mental wellness	Strongly disagree	1 (5.9%)	0	0	0	1 (2.9%)	0.262
		Disagree	1 (5.9%)	1 (7.1%)	0	0	2 (5.7%)	
		Neutral	5 (29.4%)	1 (7.1%)	0	0	6 (17.1%)	
		Agree	6 (35.3%)	5 (35.7%)	0	2 (100%)	13 (37.1%)	
		Strongly agree	4 (23.5%)	7 (50%)	2 (100%)	0	13 (37.1%)	
22	Would you like to be a mentor in mentor-mentee program	Strongly disagree	1 (5.90%)	0	1 (50%)	0	2 (5.90%)	0.331
		Disagree	0	0	0	0	0	
		Neutral	6 (35.30%)	5 (38.50%)	0	0	11 (32.40%)	
		Agree	6 (35.30%)	5 (38.50%)	1 (50%)	1 (50%)	13 (38.20%)	
		Strongly agree	4 (23.50%)	3 (23.10%)	0	1 (50%)	8 (23.50%)	
23	No. of students able to mentor	1-5	13 (76.5%)	9 (64.3%)	0	1 (50%)	23 (65.7%)	0.911
		6-10	3 (17.6%)	4 (28.6%)	2 (100%)	1 (50%)	10 (28.6%)	
		11-15	1 (5.9%)	1 (7.1%)	0	0	2 (5.7%)	
24	Does students employ mental stress to avoid work	Strongly disagree	1 (5.9%)	1 (7.1%)	0	0	2 (5.7%)	0.27
		Disagree	1 (5.9%)	1 (7.1%)	0	0	2 (5.7%)	
		Neutral	8 (47.1%)	4 (28.6%)	1 (50%)	0	13 (37.1%)	
		Agree	6 (35.3%)	6 (42.9%)	1 (50%)	0	13 (37.1%)	
		Strongly agree	1 (5.9%)	2 (14.3%)	0	2 (100%)	5 (14.3%)	
25	Is demanding schedule essential for effective training	Strongly disagree	1 (5.9%)	0	0	0	1 (2.9%)	0.873
		Disagree	2 (11.8%)	2 (14.3%)	0	1 (50%)	5 (14.3%)	
		Neutral	10 (58.8%)	6 (42.9%)	2 (100%)	1 (50%)	19 (54.3%)	
		Agree	3 (17.6%)	4 (28.6%)	0	0	7 (20%)	
		Strongly agree	1 (5.9%)	2 (14.3%)	0	0	3 (8.6%)	
26	Which contribute to enhancing mental well-being of students	Cultural or sports events	1 (5.9%)	1 (7.1%)	0	0	2 (5.7%)	0.224
		Counseling services	1 (5.9%)	1 (7.1%)	0	0	2 (5.7%)	
		Effective communication	8 (47.1%)	4 (28.6%)	1 (50%)	0	13 (37.1%)	
		Implementation of mentor-mentee	6 (35.3%)	6 (42.9%)	1 (50%)	0	13 (37.1%)	
		Healthy food availability	1 (5.9%)	2 (14.3%)	0	2 (100%)	5 (14.3%)	
		Reduced intensity of academic schedule	0	0	0	0	0	
27	Potential threat to students well-being	Behavioral changes	1 (5.9%)	1 (7.1%)	0	0	2 (5.7%)	0.257
		Mood swings	1 (5.9%)	1 (7.1%)	0	0	2 (5.7%)	
		Emotional pain	8 (47.1%)	4 (28.6%)	1 (50%)	0	13 (37.1%)	
		Declining academic performance	6 (35.3%)	6 (42.9%)	1 (50%)	0	13 (37.1%)	
		Substance abuse	0	0	0	0	0	
		Social withdrawal	0	0	0	0	0	
		Changes in eating and sleeping patterns	1 (5.9%)	2 (14.3%)	0	2 (100%)	5 (14.3%)	

Intergroup comparison by one-way ANOVA

One-way ANOVA was used to compare groups, and significant findings were confirmed using the post-hoc Bonferroni test. The one-way ANOVA revealed mean psychological well-being ratings of 3.72 for UG students, 1.90 for graduate students, and 2.94 for faculty, with UG students scoring the highest. The post-hoc Bonferroni test revealed these differences: faculty performed substantially better than PG students (mean difference 1.040, p=0.0001), but significantly worse than UG students (mean difference -0.775, p=0.0001). UG students performed much better than PG students (mean difference -1.815, p=0.0001). Overall, undergraduate students have greater levels of psychological well-being than PG students and professors (Tables 7, 8).

**Table 7:** Intergroup comparison by one-way ANOVA *p-value significant at p<0.05; one-way ANOVA was applied

Group	N	Mean	Std. Deviation	Std. Error	95% Confidence Interval for the Mean	
					Lower bound	Upper bound
Faculty	35	2.94	1.136	.192	2.55	3.33
PG	31	1.90	.597	.107	1.68	2.12
UG	284	3.72	.783	.046	3.63	3.81
Total	350	3.48	.974	.052	3.38	3.58
p-value	-	0.001	-	-	-	-

**Table 8:** Post-hoc Bonferroni for intergroup comparisons *The mean difference is significant at the 0.05 level; post-hoc Bonferroni test was applied

(I) Group	(J) Group	Mean Difference (I-J)	Std. Error	Sig.	95% Confidence Interval	
					Lower bound	Upper bound
Faculty	PG	1.040*	.200	.0001	.56	1.52
	UG	-.775*	.145	.0001	-1.12	-.43
PG	Faculty	-1.040*	.200	.0001	-1.52	-.56
	UG	-1.815*	.153	.0001	-2.18	-1.45
UG	Faculty	.775*	.145	.0001	.43	1.12
	PG	1.815*	.153	.0001	1.45	2.18

## Discussion

The study aimed to assess stress levels among UG and PG students and faculty members and their roles in assisting students with stress management.

On assessing the depression, anxiety, and stress scores, it was found that anxiety was the major issue in the population. In total, 42% of the participants reportedly had extremely severe anxiety levels, whereas depression was found to be moderate (30.9%), and surprisingly, stress was normal (57.1%). This is in agreement with the study conducted in Saudi Arabia, where it was found that even in the pre-examination stage and post-examination state, the students were reported to have high anxiety levels (63%, pre-exam; 47%, post-exam), followed by depression (43%, pre-exam; 30%, post-exam) and stress (41%, pre-exam; 30%, post-exam) [14]. These findings are similar to the study where anxiety (85%) was found to be more prevalent than depression (52%) and stress (42%) [11].

The results of the present study showed that DASS has no significant difference among different age groups and categories (UG, PG, and faculty). This suggests that academic and professional status do not have a significant impact on psychological distress levels. However, it was seen that overall, females perceive more stress than males, which is in contradiction to the previous studies done by Tangade et al. [13], Acharya [15], and Kumar et al. [16]. However, two other studies support these findings [17,18]. The potential explanation for the higher reported stress levels among female students may stem from their tendency and inclination to more openly express their thoughts and feelings. This willingness to express themselves, particularly regarding negative thoughts and feelings, might contribute to their reported higher stress levels [19].

Considering that the majority of the UG students stay with their parents and the overall nature of the family is extremely supportive, it was observed that they experience academic-related helplessness only occasionally (47.9%). This observation is supported by a study conducted by Tangade et al., which concluded that students who are day scholars experience less stress compared to those who reside in hostels [13].

The National Medical Commission (NMC) of India conducted a Pan-India study of medical college students as part of the National Task Force on Mental Health and Well-being of Medical Students. According to the research, the family environment is mostly supportive, with 13,024 (50.9%) considering it extremely supportive, 8865 (34.6%) supportive, and 1151 (4.5%) unsupportive. Some students, 3960 (15.5%), mentioned experiencing academic pressure from their families on a regular or frequent basis, and 7735 (30.2%) on occasion, but the majority of students feel supported at home. The majority of students, 14,487 (56.6%), find their academic workload reasonable yet heavy, while 5302 (20.7%) consider it too heavy, and only 391 (1.5%) believe it is light or very light. The fear of failure is a major concern among undergraduate students, with 13,204 (51.6%) agreeing or strongly agreeing that it has a detrimental impact on their performance [12]. The results of this study are consistent with those of the current research.

Based on the responses received, it became evident that first-year students perceive the academic workload to be more intensive compared to their peers in higher academic levels. The observed phenomenon is likely attributable to the challenges experienced by numerous first-year students, particularly those residing in campus accommodation. These students are still acclimating in the hostel; they confront difficulties related to assimilating into new social and physical environments, coupled with the demands of the professional curriculum and the sudden surge of academic workload.

The results indicate that there had been a gradual decrease in overall academic load in the transition from the first year to the third year, but a sudden spike was seen in the final year, which again decreased during the internship. This can be due to the sudden increase in academic programs in the final year, especially the transition from non-clinical to clinical training, patient management, and more [20]. The decrease in academic load during the internship can be due to the lack of academic burden during this period. Similar results were seen concerning social isolation. These results can bring us to the conclusion that due to an increase in academic load, students tend to socially isolate themselves. Studies show that academic pressure and loneliness have a direct association [21,22]. A study by Zhensong et al. [23] found that social connectedness and academic pressure were positively correlated.

It was seen that 47.6% of students are aware of the mental health services provided by the university they are currently studying in. However, the majority had a neutral reaction to its accessibility and rated the services as average. A study by Eisenberg et al. found that only 15% of students with mental health disorders seek help in institutes' existing mental health services. One of the factors found for this was inaccessible services [24]. This can be due to the taboo behind mental health services and lack of importance towards psychological well-being.

The study revealed that PG students face maximum stress due to the PG seat leaving bond (90.3%). Under this policy, a student cannot leave his/her course before completing it without paying a heavy fine. Although there are no substantial studies that show a direct link between the PG leaving bond and mental stress, there are few findings that suggest a potential connection.

For coping mechanisms, the primary strategies opted for by students included seeking support from loved ones (22.6%) and engaging in hobbies (19.4%). The role of social interaction and relationships was highlighted when most respondents expressed that peer interaction affect their stress levels (67.7%). A study done in 2022 by Hill et al. revealed that trainees' second most common coping mechanism was seeking social support, including family, friends, and peers [25]. This underscores the importance of personal support networks and leisure activities in managing stress. Engaging in leisure activities has been identified as a means to alleviate the adverse impacts of stressful experiences, which pose a threat to both physical and psychological well-being. Additionally, participation in such activities has been found to contribute to subjective well-being effectively [26-28]. However, some studies have shown that the more effective form of coping mechanism is problem-focused strategies rather than emotion-focused strategies [29,30]. In terms of administrative support, 64.5% of respondents indicated a need for flexible scheduling to effectively manage academic and personal pressures. Furthermore, 87.1% of participants do not turn to substance use as a coping mechanism, indicating that most individuals rely on healthier strategies to handle stress. However, a notable chunk does believe that stress levels and substance use are somewhat linked (38.7%).

A larger population believes that they face challenges concerning the thesis (90.3%) and during the submission of the thesis (54.80%). This is primarily due to the financial strain imposed by the thesis, which leads students to anticipate some type of financial assistance from the administration (38.7%), which is backed by the majority agreeing that their psychological well-being is affected by financial concerns. Notably, second-year students (72.7%) and third-year students (83.3%) faced more challenges compared to first-year students (28.6%). This suggests that thesis submission becomes increasingly challenging as students advance in their studies. This is backed by a study that showed that one of the most common stressors for students are fear of failure and finances [13]. The study done by NMC suggests that financial hardship is experienced constantly or frequently by 4744 (18.5%) students and occasionally by 8194 (32%), while the remaining feel supported and extremely supported about financial concerns [12]. The overall stress level in PG students was noted to be high (64.5%).

When assessing faculty members for their stress levels and how they help in the management of stress among students, the data show that 41.20% of teachers perceive themselves as very approachable. In total, 34.30% and 31.40% of faculty members believe it is somewhat very necessary for students to have designated breaks as they believe the college academic schedule (38.20%, p-value= 0.053) and faculty members themselves (51.4%; p-value= 0.247) creates stress for the students.

To reduce stress, the majority of the faculty members advise counseling services from college (51.4%) and try to engage with the students (76.50%) to alleviate their stress levels. Although the faculty had discussions with students about mental wellness (77.1%), the major chunk does not share examples to help the students overcome challenges (91.4%). Overall, 51.4% of faculties believe that they can influence students' psychological well-being and would like to mentor students (38.20%) for their betterment under the mentor-mentee program, thus agreeing that the implementation of the mentor-mentee program (37.1%) and effective communication (37.1%) can enhance the psychological well-being of the students.

Limitations of the study

There are some limitations to the study. Given that the present study was a cross-sectional study conducted in one region, it cannot necessarily represent all of India's dental students. The self-reporting nature of the data collection can be subjected to biases such as memory, social desirability, and confidentiality that may affect our findings. The study revealed a higher number of UG than PG students, as the intake of seats is higher in UGs than in PGs in India. Additionally, there was a significantly larger number of female respondents as compared to males, as they preferred to opt for dentistry over males. These findings could potentially impact the conclusions, as they indicate that females may be more susceptible to stress than males. Despite these limitations, this study is one of the first to assess the faculty members' stress and their method of helping students with stress management, along with trying to assess the potential environmental risk factors for psychological well-being.

## Conclusions

The study discovered that females sense stress more than males. Most undergraduate students live with supportive families and only face academic difficulties in rare instances. Academic work is more demanding for first-year students than it is for their higher-education classmates. Overall academic load progressively reduces from the first to the third year but then considerably increases in the last year before decreasing during internships. As the academic load grows, students tend to distance themselves socially. Participating in leisure activities can help reduce the detrimental effects of stress on physical and psychological health. Faculty members encourage college counseling programs to help students deal with stress.

## Appendices

Questionnaire 1

Depression, Anxiety, and Stress Scale (DASS-21)

The DASS-21 is a shortened version of the DASS-42, a self-report scale used to assess the negative emotional states of depression, anxiety, and stress. The DASS three scales have been demonstrated to have good internal consistency and to provide significant discriminations. Thus, they should fulfill the demands of both researchers and clinicians who want to evaluate present status or change in state over time (for example, during therapy). This scale is appropriate for both clinical and nonclinical contexts. Please read each statement and select a number (0, 1, 2, or 3) indicating how much the statement applies to you over the last week. There are no correct or incorrect replies. Do not spend too much time on any statement.

1. I found it difficult to wind down.

a)     Almost always

b)     Often

c)     Sometimes

d)     Never

2. I was conscious of dryness in my mouth.

a)     Almost always

b)     Often

c)     Sometimes

d)     Never

3. I couldn't seem to have any positive feelings at all.

a)     Almost always

b)     Often

c)     Sometimes

d)     Never

4. I experienced breathing difficulty (excessively rapid breathing, breathlessness in the absence of physical exertion)

a)     Almost always

b)     Often

c)     Sometimes

d)     Never

5. I found it tough to take the initiative to do things.

a)     Almost always

b)     Often

c)     Sometimes

d)     Never

6. I tended to overreact to circumstances.

a)     Almost always

b)     Often

c)     Sometimes

d)     Never

7. I experienced trembling (for example, in my hands).

a)     Almost always

a)     Often

b)     Sometimes

c)     Never

8. I felt like I was exerting a lot of nervous energy.

a)     Almost always

a)     Often

b)     Sometimes

c)     Never

9. I was concerned about circumstances in which I may panic and make a fool of myself.

a)     Almost always

a)     Often

b)     Sometimes

c)     Never

10. I thought there was nothing to look forward to.

a)     Almost always

a)     Often

b)     Sometimes

c)     Never

11. I felt myself becoming agitated.

a)     Almost always

a)     Often

b)     Sometimes

c)     Never

12. I found it difficult to relax

a)     Almost always

b)     Often

c)     Sometimes

d)     Never

13. I felt depressed and blue.

a)     Almost always

a)     Often

b)     Sometimes

c)     Never

14. I was intolerant of anything that prevented me from continuing with what i was doing.

a)     Almost always

a)     Often

b)     Sometimes

c)     Never

15. I felt I was close to panic

a)     Almost always

b)     Often

c)     Sometimes

d)     Never

16. I couldn’t get excited about anything.

a)     Almost always

a)     Often

b)     Sometimes

c)     Never

17. I thought I wasn't worth much as a person.

a)     Almost always

a)     Often

b)     Sometimes

c)     Never

18. I felt like I was somewhat sensitive.

a)     Almost always

b)     Often

c)     Sometimes

d)     Never

19. I was aware of the action of my heart in the absence of physical exertion (for example, awareness of heart rate rise, heart skipping a beat).

a)     Almost always

a)     Often

b)     Sometimes

c)     Never

20. I felt afraid for no apparent reason.

a)     Almost always

a)     Often

b)     Sometimes

c)     Never

21. I felt that my life was meaningless.

a)     Almost always

b)     Often

c)     Sometimes

d)     Never

Questionnaire 2

Assessing the Psychological Well-Being Among Dental Students and Faculty: A College-Wide Survey

Questionnaire

Thank you for participating in this survey. Your feedback is highly valued. Please take note of the following:

Your responses are anonymous.

Your responses will remain confidential and only be used for this survey.

Data collected will be used for research and analysis and will not be shared publicly.

While we have taken security measures, please be aware that no method of transmission over the internet is entirely secure.

By proceeding with this survey, you must acknowledge that you have read, understood, and agreed to the above disclaimer.

Consent

1.      Do you give consent to be a part of this study?

a.      Yes

b.      No

Demographic Details

1.      Name

2.      Age

3.      Gender

a.      Male

b.      Female

c.      Others

d.      Prefers not to say

4.      College Name

5.      Please select the relevant survey category.

a.      Faculty

b.      Post-Graduate

c.      Under-Graduate

Section 1: Faculty and Administrator

1.      Position you currently hold

a.      Faculty

b.      Administrator (Dean, Principal, HOD)

2.      Years of teaching experience:

a.      < less than 10 years

b.      10-20 years

c.      20-30 years

d.      More than 30 years

3.      How would you describe your level of approachability to your student? Please rate it on a scale of 1 to 5, wherein,

1 = Very Unapproachable,

2 = Somewhat Unapproachable,

3 = Neutral,

4 = Somewhat Approachable,

5 = Very Approachable

a.      1

b.      2

c.      3

d.      4

e.      5

4.      Do you think students should have designated leisure time/breaks to unwind and relax? Please rate it on a scale of 1 to 5, wherein,

1 = Very unnecessary

2 = Somewhat unnecessary

3 = Neutral

4 = Somewhat necessary

5 = Very necessary

a.      1

b.      2

c.      3

d.      4

e.      5

5.      Do you think the academic schedule and environment at your college create stress for students? Please rate it on a scale of 1 to 5, wherein,

1 = Extremely Stressful,

2 = Stressful,

3 = Moderately Stressful,

4 = Slightly Stressful,

5 = Not Stressful

a.      1

b.      2

c.      3

d.      4

e.      5

6.      Do you believe faculty members sometimes contribute to unnecessary mental stress for students?

a.      Yes

b.      No

7.      Are you aware of any counselling services in your college for the students?

a.      Yes

b.      No

8.      If yes, have you advised these facilities to any of your students?

a.      Yes

b.      No

9.      If yes, in past 5 years, how many students have you referred to this counselling services?

a.      None

b.      1-3

c.      4-5

d.      >5

10.   What actions do you implement to alleviate the mental stress experienced by your students?

a.      Guaranteeing sufficient rest and nutrition for them

b.      Engaging with them

c.      Additional assistance in teaching

11.   Have you encountered any difficulties in handling instances of depression among medical students?

a.      Yes

b.      No

12.   Have you ever shared examples/instances which might help your students to overcome challenges?

a.      Yes

b.      No

13.    Does your college have a well-defined system in place to address or handle cases of depression among students?

a.      Yes

b.      No

14.   Over the last two years, how many extracurricular events, wellness workshops, or recreational activities have you attended/helped organize?

a.      None

b.      1-3

c.      4-5

d.      >5

15.   Do you believe it is important to regularly organize extracurricular events, wellness workshops, or recreational activities at your college? Please rate it on a scale of 1 to 5, wherein,

1 = Very unnecessary

2 = Somewhat unnecessary

3 = Neutral

4 = Somewhat necessary

5 = Very necessary

a.       1

b.      2

c.      3

d.      4

e.      5

16.   Have you ever had discussions with your students about mental wellness and stress in college?

a.      Yes

b.      No

17.   Do you effectively communicate constructive ideas to enhance the wellness and learning experience of your students to your superiors? Please rate it on a scale of 1 to 5, wherein,

1 = Never

2 = Rarely

3 = Sometimes

4 = Always

5 = Often

a.      1

b.      2

c.      3

d.      4

e.      5

18.   Does the institution have a well-defined protocol in place to address instances of discrimination or bias that students may encounter based on factors such as their financial status, social background, cultural background, or academic history?

a.      Yes

b.      No

19.   In the past month, which of the following methods have you utilized to nurture your mental health and overall well-being?

a.      Socialized with friends 

b.      Spent time with family 

c.      Listened to music 

d.      Enjoyed a favorite meal 

e.      Exercised 

f.       Connected with nature 

g.      Prioritized sleep 

h.      Established or maintained a routine 

i.       Applied time management techniques 

j.       Consulted with a counselor or healthcare professional 

k.      k. Engaged in spiritual contemplation 

l.       Engaged in prayer or connected with a religious community 

m.    Meditated 

n.      Other activities

20.   Rate your stress levels at your workplace on a scale of 1 to 5, wherein,

1 = Not Stressful,

2 = Slightly Stressful,

3 = Moderately Stressful,

4 = Stressful,

5 = Extremely Stressful

a.      1

b.      2

c.      3

d.      4

e.      5

21.   Do you believe you have the ability to influence your student's psychological well-being? Please rate it on a scale of 1 to 5, wherein,

1 = Strongly Disagree

2 = Disagree

3 = Neutral

4 = Agree

5 = Strongly Agree

a.      1

b.      2

c.      3

d.      4

e.      5

22.   Do you believe that your superiors will support your efforts to launch activities aimed at enhancing mental wellness among your students? Please rate it on a scale of 1 to 5, wherein,

1 = Strongly Disagree

2 = Disagree

3 = Neutral

4 = Agree

5 = Strongly Agree

a.      1

b.      2

c.      3

d.      4

e.      5

23.   If there is mentor-mentee program, would you like to be a mentor for a student? Please rate it on a scale of 1 to 5, wherein,

1 = Strongly Disagree

2 = Disagree

3 = Neutral

4 = Agree

5 = Strongly Agree

a.      1

b.      2

c.      3

d.      4

e.      5

24.   In your opinion, how many students would you be able to mentor during the course of their program?

a.      1-5

b.      6-10

c.      11-15

25.   Do you believe students employ mental stress as a means to avoid work or as a justification for avoiding work? Please rate it on a scale of 1 to 5, wherein,

1 = Strongly Disagree

2 = Disagree

3 = Neutral

4 = Agree

5 = Strongly Agree

a.      1

b.      2

c.      3

d.      4

e.      5

26.   Do you believe that a demanding schedule and psychological pressure are essential for the effective training of medical professionals? Please rate it on a scale of 1 to 5, wherein,

1 = Strongly Disagree

2 = Disagree

3 = Neutral

4 = Agree

5 = Strongly Agree

a.      1

b.      2

c.      3

d.      4

e.      5

27.   Which of the following actions do you believe will contribute to enhancing mental well-being among students?

a.      Reduced intensity of academic schedule 

b.      Increased number of off days 

c.      Arrangement of cultural or sports events 

d.      Ensuring healthy food availability on campus 

e.      Improved residential facilities in the college 

f.       Availability of counseling services 

g.      Allocation of space on campus for sports activities 

h.      Emphasis on effective communication with faculty 

i.       Implementation of mentor-mentee programs 

j.       Provision of financial support or special scholarships for students with limited resources 

k.      Other (please specify)

28.   Which of the following indicates potential threats to mental health and may occur before attempts at self-harm? (Select the applicable options)

a.      Withdrawal in which students suddenly avoid social situations and isolate themselves from others.

b.      Substance abuse wherein there is a sudden increase in alcohol or drug consumption.

c.      Expressing unbearable emotional or physical pain.

d.      Behavioral changes such as making plans or researching methods of suicide, withdrawing from social circles, saying farewells, giving away meaningful/important belongings, or creating a will.

e.      Displaying extreme mood swings.

f.       Experiencing significant changes in eating or sleeping patterns.

g.      Demonstrating declining academic performance and deteriorating personal hygiene.

Section 2: Post-Graduate Dental Students (MDS)

1.      Year

a.      First

b.      Second

c.      Third

2.      Marital Status

a.      Single

b.      Married

c.      In a relationship

d.      Divorced

e.      Others (please specify)

3.      Family income

a.      Rs. 1-10 lakh/annum

b.      Rs. 11-20 lakh/annum

c.      Rs. 21-30 lakh/annum

d.      Rs. 41-50 lakh/annum

4.      How pleased are you with the overall amenities offered by the college? Please rate it on a scale of 1 to 5, wherein,

1 = extremely poor,

2 = poor,

3 = Satisfied,

4 = Good,

5 = Very Good

a.      1

b.      2

c.      3

d.      4

e.      5

5.      Do you feel stressed about the PG seat leaving bond in your college?

a.      Yes

b.      No

6.      Who do students at your hospital/college typically experience the most problems with?

a.      Postgraduate seniors

b.      Faculty members

c.      Senior residents

d.      Admin/Others

7.      Support from Advisor/Teacher - How satisfied are you with the support provided by your Guide/teacher?

a.      Adequate

b.      Inadequate

c.      Needs Improvement

8.      How do you usually cope with stress from academic demands? (select the relevant options)

a.      Seeking assistance from loved ones 

b.      Seeking guidance from a counselor or therapist 

c.      Participating in physical activities or sports 

d.      Using relaxation techniques (like yoga) 

e.      Pursuing hobbies or listening to music 

f.       Using substances (please specify if applicable) 

g.      Taking care of oneself 

h.      Postponing or delaying stress-inducing tasks 

i.       Engaging in self-harm (please specify, if comfortable) 

j.       Other methods (please specify)

9.      Are you worried about how your family or spouse would respond if you discussed your problems with them?

a.      Yes

b.      No

10.   Have interactions with peers affected your stress levels?

a.      Yes

b.      No

11.   For an effective way to address bullying, what support mechanism do you think should be improved?

a.      Counselling services

b.      Anti-ragging committees

c.      Hotline numbers

d.      Others (please specify)

12.   How has your marriage or relationship impacted your stress levels as a postgraduate?

a.      Adversely

b.      Helps me cope

c.      Not applicable (unmarried/not in relationship)

13.   To manage your relationship commitments, what sort of support do you expect from the administration?

a.      Counselling services

b.      Communication workshops

c.      Flexible scheduling

d.      Other, please specify

14.   To cope with the stress, have you ever felt pressured to use any sort of substance such as alcohol, tobacco, or drugs?

a.      Yes

b.      No

15.   How do you view the connection between stress levels and substance use among your peers?

a.      Strongly linked

b.      Somewhat linked

c.      Not linked

d.      Workload

16.   Is your psychological well-being affected by your course?

a.      Yes

b.      No

17.   For your thesis, have you faced any challenges?

a.      Yes

b.      No

18.   How much stress do you feel due while submitting your thesis?

a.      Less

b.      Moderate

c.      High

d.      Severe

19.   How would you like the administration to help you with the thesis?

a.      Removal of thesis

b.      More time

c.      Support financially

d.      Guidance of faculty

e.      Other, please mention

20.   Select all the appropriate reasons for your mental health troubles.

a.      Stress due to academics

b.      Ragging by seniors

c.      Issues from administration

d.      Family Issues

e.      Relationship issues

f.       Financial issues

g.      Psychiatric Illness

h.      Cyberbullying

i.       Sexual harassment

j.       Sexual orientation

k.      Others, please specify

21.   What challenges do you feel you might face if you seek help from a therapist/counselor?

a.      Stigma

b.      Discrimination

c.      Confidentiality concerns

d.      Impact on future job prospects

e.      Licensing issues

f.       Other

22.   How would you rate your overall stress levels as a post-graduate student?

a.      No stress

b.      Low Stress

c.      Moderate Stress

d.      High Stress

e.      Very High Stress

23.   Effective self-care practices- what self-care practices or strategies have you found most effective in managing stress? (Select 3 most appropriate answers)

a.      Keeping a journal 

b.      Spending time with family and friends 

c.      Talking to friends and relatives 

d.      Going on trips 

e.      Pursuing hobbies 

f.       Taking part in sports and games 

g.      Practicing meditation 

h.      Doing yoga 

i.       Attending stress management workshops 

j.       Engaging in spiritual practices 

k.      Other activities

24.   Is your psychological well-being affected by the financial concerns?

a.      No

b.      Yes

25.   How do you cope with financial stress?

a.      Budgeting

b.      Seeking financial assistance like loans

c.      Other

Section 3: Under-Graduate Dental Students (BDS)

1.      Year

a.      First

b.      Second

c.      Third

d.      Fourth

e.      Intern

2.      Is the college you currently are studying in the state where you belong from?

a.      Yes

b.      No

3.      Where do you reside?

a.      Parent(s)

b.      Relatives

c.      Hostel

d.      Own Apartment/Shared Apartment

e.      Other (please specify)

4.      Family income, cumulative

a.      Rs. 1-10 lakh/annum

b.      Rs. 11-20 lakh/annum

c.      Rs. 21-30 lakh/annum

d.      Rs. 41-50 lakh/annum

e.      Rs. 51 lakh and above/annum

5.      Number of siblings

a.      0

b.      1

c.      2

d.      3

e.      4+

6.      Please rate the overall supportive nature of your family on a scale of 1 to 5, wherein,

1 = extremely unsupportive,

2 = unsupportive,

3 = neutral,

4 = supportive,

5 = extremely supportive.

a.      1

b.      2

c.      3

d.      4

e.      5

7.      How often do you experience any of the following in your family environment? On a scale of 1 to 5, wherein,

1 = always,

2 = often,

3 = sometimes,

4 = supportive,

5 = extremely supportive

a.      Pressure of academics from family.

                                                    i.     1

                                                  ii.     2

                                                 iii.     3

                                                 iv.     4

                                                   v.     5

b.      Financial strain in the family.

                                                    i.     1

                                                  ii.     2

                                                 iii.     3

                                                 iv.     4

                                                   v.     5

8.      Please rate the overall load of your academics.

a.      Very Stressful

b.      Stressful

c.      Manageable

d.      Easy

e.      Very Easy

9.      How frequently do you feel helpless by academic demands?

a.      Always

b.      Often

c.      Sometimes

d.      Rarely

e.      Never

10.   Please rate the following statements on a scale of 1 to 5, wherein,

1 = Strongly Disagree,

2 = Disagree,

3 = Neutral,

4 = Agree,

5 = Strongly Agree

a.      My skills and knowledge are assessed appropriately by exams.

                                                    i.     1

                                                  ii.     2

                                                 iii.     3

                                                 iv.     4

                                                   v.     5

b.      My academic performance is adversely affected due to fear of failure.

                                                    i.     1

                                                  ii.     2

                                                 iii.     3

                                                 iv.     4

                                                   v.     5

c.      I have difficulty balancing my personal well-being and academics

                                                    i.     1

                                                  ii.     2

                                                 iii.     3

                                                 iv.     4

                                                   v.     5

11.   Do the library's operating hours and amenities support your study requirements?

a.      Yes

b.      No

c.      Somewhat

12.   Do you believe the college has sufficient measures in place to prevent and handle incidents of ragging?

a.      Yes

b.      No

c.      Unsure

13.   How much pressure do your finances (tuition, living expenses, etc.) place on you? Please rate it on a scale of 1 to 5, where:

1 = extremely stressful

2 = significantly stressful

3 = moderately stressful

4 = Slightly stressful

5 = Not stressful

a.      1

b.      2

c.      3

d.      4

e.      5

14.   How frequently do you experience feelings of loneliness or social isolation?

a.      Never

b.      Rarely

c.      Sometimes

d.      Often

e.      Always

15.   Have you ever received a diagnosis for any mental health conditions?

a.      Anxiety

b.      Depression

c.      Attention deficit disorders

d.      Eating disorders

e.      Bipolar disorder

f.       Other

g.      None of the above

16.   Rate the level of support you feel from:

Please rate it on a scale of 1 to 5 wherein:

1 = extremely unsupportive

2 = unsupportive

3 = neutral

4 = supportive

5 = extremely supportive

Faculty/Mentors

                                                    i.     1

                                                  ii.     2

                                                 iii.     3

                                                 iv.     4

                                                   v.     5

17.   Does your college offer an official program of mentor-mentees?

a.      Yes

b.      No

c.      Unsure

18.   How effective is having a mentor and meeting them regularly?

a.      Very effective

b.      Somewhat effective

c.      Neutral

d.      Somewhat ineffective

e.      Very ineffective

19.   Are you at ease with accessing mental health assistance like counseling, therapy, or mentorship if necessary?

a.      Very uncomfortable

b.      Somewhat Uncomfortable

c.      Neutral

d.      Somewhat comfortable

e.      Very comfortable

20.   Do you know about any mental health support services offered at your dental college?

a.      Yes

b.      No

c.      Unsure

21.   If yes, please rate these features of the support services in your college:

a.      Accessibility

                                                   i.     Very inaccessible

                                                  ii.     Somewhat inaccessible

                                                  iii.     Neutral

                                                  iv.     Somewhat accessible

                                                  v.     Accessible

b.      Quality

                                                  i.     Very poor

                                                  ii.     Poor

                                                 iii.     Average

                                                 iv.     Good

                                                 v.     Excellent
